# Patient Comfort in Modern Computed Tomography: What Really Counts

**DOI:** 10.3390/tomography8030113

**Published:** 2022-05-23

**Authors:** Julius Henning Niehoff, Andreas Heuser, Arwed Elias Michael, Simon Lennartz, Jan Borggrefe, Jan Robert Kroeger

**Affiliations:** 1 Department of Radiology, Neuroradiology and Nuclear Medicine, Johannes Wesling University Hospital, Ruhr University Bochum, 44801 Bochum, Germany; andreas.heuser@ruhr-uni-bochum.de (A.H.); arwed.michael@muehlenkreiskliniken.de (A.E.M.); jan.borggrefe@muehlenkreiskliniken.de (J.B.); janrobert.kroeger@muehlenkreiskliniken.de (J.R.K.); 2 Institute for Diagnostic and Interventional Radiology, Faculty of Medicine and University Hospital Cologne, University of Cologne, 50937 Cologne, Germany; simon.lennartz@uk-koeln.de

**Keywords:** computed tomography, patient comfort, photon-counting CT

## Abstract

Background: The purpose of the present study is to evaluate the patient comfort during CT examinations with a modern CT scanner (Photon-Counting CT (PCCT)) and to compare the perceived patient comfort with a standard CT scanner. Methods: A total of 157 patients participated in this study and completed a questionnaire on their subjective perceptions after their CT examination. The following aspects of comfort were rated on a 5-point Likert scale: (1) claustrophobia in general and during the examination, (2) the effort to lie down and to get up from the CT table, (3) the speed and comfort of the CT table, (4) the difficulty of holding the breath during the CT scan, and (5) the communication during the examination. Results: Patients rated the modern CT scanner significantly better in terms of speed and comfort of the CT table and in terms of difficulty of holding their breath during the CT scan. The answers regarding the other aspects of comfort did not reveal significant differences. When asked for a comparison, patients who did perceive a difference between both scanners rated the modern CT scanner as more comfortable in all aspects of comfort. Conclusions: The survey did not reveal any major deficits in terms of comfort on the standard CT scanner. However, patients perceived the structural changes positively and appreciated the comfort of a modern CT scanner.

## 1. Introduction

Advances in medicine are usually understood as relevant basic scientific findings or a significant improvement in therapy outcomes. In contrast, advances in patient comfort are discussed less frequently, although patient comfort is a highly important aspect of medical care. Patients requiring medical treatment or diagnostics usually do not feel well mentally or physically. Thus, it is important to offer the best possible comfort during an examination or a treatment in order to achieve optimal clinical results. This applies to both the personal contact between patients and medical staff as well as to the use of modern technologies. In this context, the European Society of Radiology (ESR) has coined the term value-based radiology. By listing and discussing ideas, the ESR endeavors to define, evaluate, and enhance value in delivering radiology services to patients [1]. 

In 2021, the first clinical CT scanner that makes use of a photon-counting detector with quantum technology was introduced (Photon-Counting CT, PCCT, Naeotom Alpha, Siemens Healthineers, Erlangen, Germany). This innovative technology can provide great advances in image quality and resolution [2,3]. The first published studies underline the potential of this novel scanner [4,5]. Furthermore, by acquiring a spectral dataset, this novel CT system offers a variety of postprocessing capabilities, similar to the well-established dual-energy CT systems [6]. For example, it is possible to reconstruct virtual non-contrast (VNC) images from contrast-enhanced CT scans. These VNC images could potentially replace true non-contrast CT scans, which, in turn, would reduce the overall examination time as well as the radiation dose [7,8,9,10]. In summary, the novel photon-counting detector could improve imaging capabilities. However, these improvements are unlikely to have a major impact on the patient experience during CT examinations. It might be more important to patients that the innovative detector technology of the PCCT is combined with a high-end scanner platform that offers a number of structural innovations compared to standard CT scanners that can potentially improve patient comfort. These features include a CT table that can be lowered to a minimum height of 38 centimeters, which may make it easier to lie down and get up. The diameter of the gantry measures 82 centimeters. At the same time, however, the gantry is comparably deep, which may have an influence on patients who have claustrophobia. The speed of the CT table can be increased to a maximum of 737 millimeters/second. The scan time and the time in which patients have to hold their breath is relatively short, which may be beneficial as patients sometimes struggle to hold their breath during the entire CT scan. In addition to audible instructions, patients can now also visually follow the breathing instructions. This is accomplished by two small monitors that are in the field of vision of patients in the supine position. The instructions are visualized with simple pictograms. It is important to note that these patient comfort-related features are not necessarily unique for the PCCT. Other modern CT scanners might also be equipped with these features. 

Patient comfort during radiological examinations is the subject of just a small number of studies. However, most of these studies evaluate the patient experience during high field MR imaging [11,12,13,14,15,16]. Furthermore, there are studies that evaluate the patient comfort during positron emission tomography/magnetic resonance (PET/MR) and positron emission tomography/computed tomography (PET/CT) [17]. Further studies compared the comfort of cone-beam breast CT and digital mammography or the influence of contrast material temperature on patient comfort and image quality [18,19,20].

The purpose of the present study is to evaluate the patient experience during CT examinations with a dedicated focus on current advances and their effect on patient comfort beyond the marketing of single manufacturers. For that matter, we compared the perceived patient comfort in different categories in CT examinations performed with a modern, recently released CT Scanner (PCCT, Naeotom Alpha, Siemens Healthineers, Erlangen, Germany) and with a standard CT scanner (SCT, Somatom Sensation 40, Siemens Healthineers, Erlangen, Germany).

## 2. Materials and Methods

### 2.1. Scanner Characteristics

The examinations were carried out either on the recently introduced PCCT (Naeotom Alpha, Siemens Healthineers, Erlangen, Germany) or on a standard CT scanner (SCT, Somatom Sensation 40, Siemens Healthineers, Erlangen, Germany). The characteristics of both scanners are compared in Table 1.

### 2.2. Study Population

A total of 157 patients who underwent a CT scan between September and November 2021 agreed to participate in this prospective single-center study and completed a questionnaire on their subjective perceptions after their CT examination. Of those, 113 patients (57 male and 56 female) were examined with the PCCT; 44 patients (22 male and 22 female patients) were examined with the SCT. The study received ethics board approval and was conducted in accordance with current good clinical practice (GCP) guidelines.

### 2.3. Questionnaire

The patients were asked to complete questionnaires immediately after their CT examination. The questionnaires focused on the perception of comfort during the CT scan. Five different aspects of comfort had to be evaluated on a five-point Likert scale: (1) claustrophobia in general and during the examination, (2) the effort to lie down and get up from the CT table, (3) the speed and comfort of the CT table, (4) the difficulty of holding the breath during the CT scan, and (5) the communication during the examination. After an examination with the PCCT, patients who had experienced the SCT scanner in the past were additionally asked to compare both scanners. In addition, we discuss the impressions expressed by the patients regarding the individual aspects of comfort.

### 2.4. Statistics

Established software packages were used for the statistical analysis (SPSS Statistics 28, IBM, Armonk, NY, USA; Excel 2016, Microsoft, Redmond, WA, USA; R Core Team (2021). R: A language and environment for statistical computing. R Foundation for Statistical Computing, Vienna, Austria; R Studio Version 1.4.1106). If not stated otherwise, all data are presented as mean ± standard deviation from the mean (SD). The significance of differences between PCCT and SCT was tested by the Mann–Whitney U test, and *p*-values ≤ 0.05 were considered statistically significant. The distribution of answers for the PCCT and SCT is visualized as horizontal stacked bar charts. Waffle charts are used to show the distribution of answers regarding the comparison between both scanners by individual patients.

## 3. Results

### 3.1. Patient Population

A total of 113 patients (57 male and 56 female) completed the questionnaire after their diagnostic CT examinations with the PCCT. The mean age of these patients was 66.8 years (range 24–91 years). Ninety-eight patients (87%) had a CT scan of the chest, the abdomen, or a combination of both (chest and abdomen). Ten patients (9%) had a CT scan of the head. The majority of patients (*n* = 64, 57%) who were interviewed after their CT examination with the PCCT declared to have experienced CT examinations with the SCT in the past and were, therefore, able to compare both CT scanners. Most of the patients (73%) were able to report on their experience from ≤4 CT examinations. Twenty-five patients (22%) had 5–10 CT examinations, and five patients (4%) had more than 10 CT examinations in the past.

A total of 44 patients (22 male and 22 female patients) completed the questionnaire after their diagnostic CT examinations with the SCT scanner. The mean age of these patients was 62.8 years (range 18–84 years). Twenty-six patients (59%) had a CT scan of the chest, the abdomen, or a combination of both (chest and abdomen). Thirteen patients (30%) had a CT scan of the head. The majority of patients (*n* = 25, 57%) who were interviewed after their CT examination with the SCT have experienced CT examinations in the past. Most of the patients (86%) were able to report on their experience from ≤4 CT examinations. Two patients (5%) had 5–10 CT examinations, and three patients (7%) had more than 10 CT examinations in the past.

### 3.2. Claustrophobia

Of all patients who were examined with the PCCT, 33.6% declared that they generally suffer from mild or severe claustrophobia. However, only 0.9% of all patients stated that they felt claustrophobic during the examination, whereas the majority of patients (99.1%) said that they did not encounter claustrophobic feelings.

**Figure 1:**
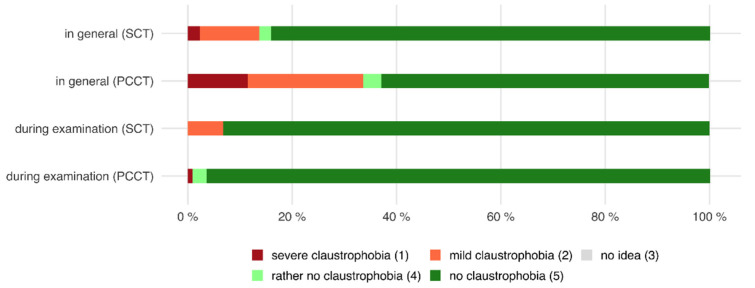
Horizontal stacked bar chart showing the distribution of answers regarding the perceived feeling of claustrophobia in general and during the CT examination. Although the percentage of patients declaring that they generally have claustrophobia before the CT examination is considerably high (33.6% PCCT; 13.6% SCT), only a few patients stated that they felt claustrophobic during the examination (PCCT 4.94 ± 0.4 versus SCT 4.80 ± 0.8, *p* = 0.369).

Likewise, 13.6% of all patients who were examined with the SCT declared that they generally suffer from mild or severe claustrophobia. After the examination, 6.8% of all patients stated that they experienced mild claustrophobia, whereas the majority (93.2%) did not have claustrophobic feelings at all (see also Figure 1).

**Figure 2:**
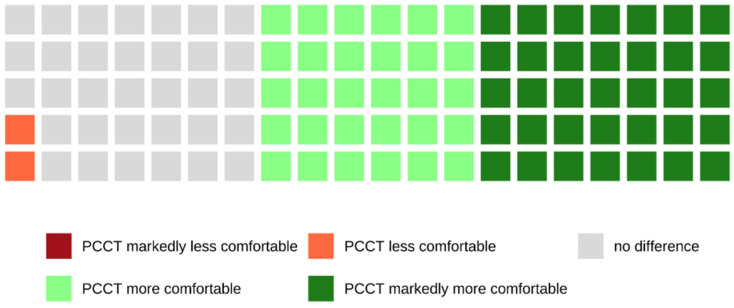
Waffle plot showing the comparison of both CT scanners in terms of claustrophobia where 65.1% of patients rated the examination with the PCCT as more or markedly more comfortable compared to an examination SCT.

Of all patients who were examined with the PCCT and who were able to make a comparison between both scanners because of their previous CT examinations with the SCT, 65.1% rated the examination with the PCCT as more or markedly more comfortable, whereas 33.3% did not notice a difference between both scanners (see also Figure 2).

### 3.3. Effort to Lie Down and Get Up from the CT Table

Of all patients who were examined with the PCCT, 88.1% declared that it was easy or very easy to lie down and get up from the CT table, whereas 11.9% of all patients found it difficult or very difficult (mean 4.21 ± 1.0). 

**Figure 3:**
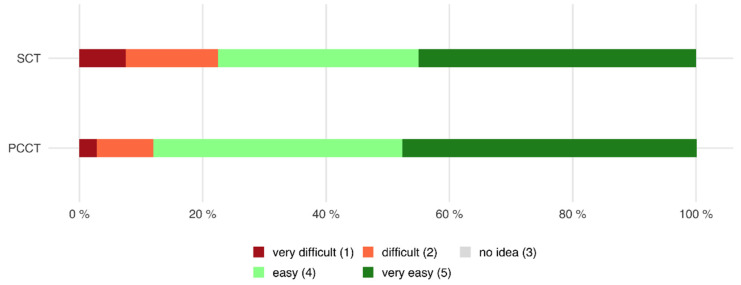
Horizontal stacked bar chart showing the perceived effort to lie down and get up from the CT table. Overall, only very few patients experienced difficulties lying down and getting up on both scanners, and there was no significant difference (PCCT 4.21 ± 1.0 versus SCT 4.04 ± 1.2, *p* = 0.447).

Of all patients that were examined with the SCT, 77.5% stated that it was easy or very easy, whereas 22.5% found it difficult or very difficult to lie down and get up from the CT table (mean 4.04 ± 1.2, *p* = 0.447 compared to PCCT, see also Figure 3).

**Figure 4:**
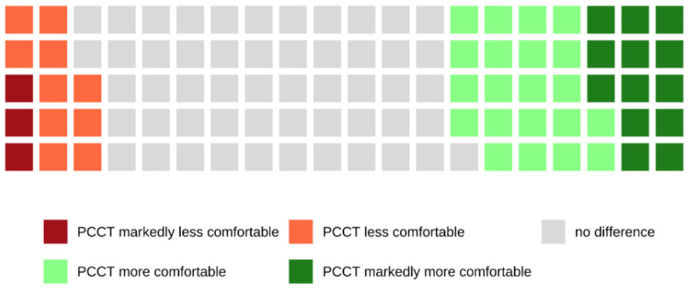
Waffle chart showing the comparison of both CT scanners in terms of effort to lie down and get up from the CT table. Most patients did not perceive any difference between the PCCT and the SCT (53.2%), and 33.9% of patients rated the examination with the PCCT as more or markedly more comfortable.

Of all patients who were examined with the PCCT and who were able to make a comparison between both scanners because of their previous CT examinations with the SCT, 33.9% rated the examination with the PCCT as more or markedly more comfortable, whereas 53.2% did not notice a difference between both scanners (see also Figure 4).

### 3.4. Speed and Comfort of the CT Table

Of all patients who were examined with the PCCT, 98.2% declared that the speed of the table was rather not too fast or not too fast and that the CT table was comfortable, whereas only 1.8% of all patients declared the opposite (mean 4.93 ± 0.4).

**Figure 5:**
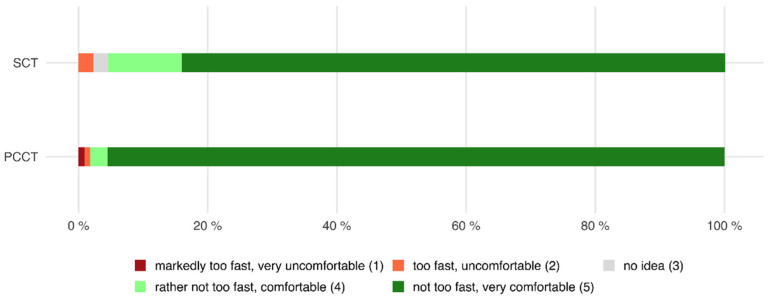
Horizontal stacked bar chart showing the perceived speed and comfort of the CT table. Although there was no difference in the mean, the different distribution led to a significant difference between the perceived speed and comfort between the PCCT and the SCT (PCCT 4.93 ± 0.4 versus SCT 4.93 ± 0.3, *p* = 0.019).

Of all patients who were examined with the SCT, 95.5% stated that the speed of the table was rather not too fast or not too fast and that the CT table was comfortable, whereas 2.3% found the opposite (mean 4.93 ± 0.3, *p* = 0.019 compared to PCCT, see also Figure 5).

**Figure 6:**
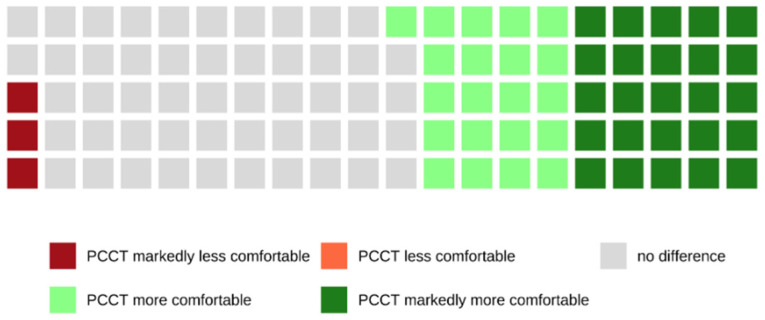
Waffle chart showing the comparison of both CT scanners in terms of speed and comfort of the CT table. Most patients (50.8%) did not notice a difference between both scanners, but 46.0% of the patients perceived the PCCT as more or markedly more comfortable.

Of all patients who were examined with the PCCT and who were able to make a comparison between both scanners because of their previous CT examinations with the SCT, 46.0% rated the examination with the PCCT as more or markedly more comfortable, whereas 50.8% did not notice a difference between both scanners (see also Figure 6).

### 3.5. Holding Breath

Of all patients who were examined with the PCCT, 97.0% declared that the time in which they had to hold their breath was rather not too long or not too long, whereas only 2.0% of all patients declared that it was too long or markedly too long (mean 4.9 ± 0.5).

**Figure 7:**
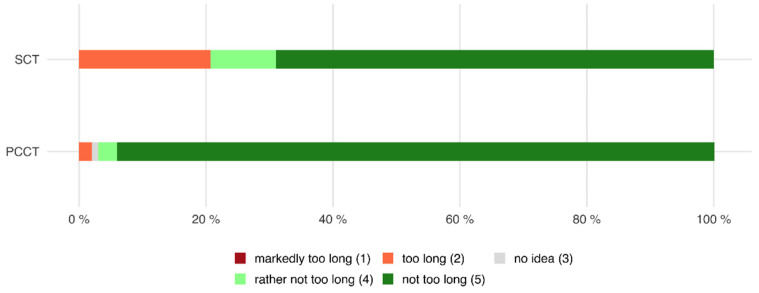
Horizontal stacked bar chart showing the perceived time to hold the breath. Patients had significantly less difficulty holding their breath when examined with the PCCT compared to the SCT (PCCT 4.9 ± 0.5 versus SCT 4.36 ± 1.2, *p* < 0.001).

Of all patients who were examined with the SCT, 79.3% stated that the time in which they had to hold their breath was rather not too long or not too long, whereas 20.7% found that the time was too long or even markedly too long (mean 4.36 ± 1.2, *p* < 0.001 compared to PCCT, see also Figure 7).

**Figure 8:**
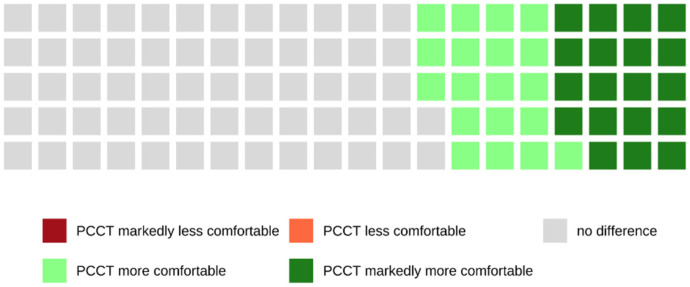
Waffle chart showing the comparison of both CT scanners in terms of holding the breath. Most patients (61.4%) did not perceive a difference between both scanners, but 38.6% of patients perceived the breath-hold duration as more or markedly more comfortable when examined with the PCCT.

Of all patients who were examined with the PCCT and who were able to make a comparison between both scanners because of their previous CT examinations with the SCT, 38.6% rated the examination with the PCCT as more or markedly more comfortable, whereas 61.4% did not notice a difference between both scanners (see also Figure 8).

### 3.6. Communication

Of all patients who were examined with the PCCT, 97.0% declared that the communication during the CT examination was good or very good, whereas only 2.0% of all patients declared that the communication was bad or very bad (mean 4.77 ± 0.5).

**Figure 9:**
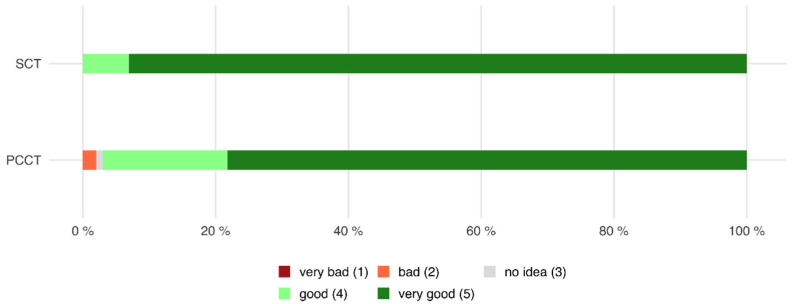
Horizontal stacked bar chart showing the perceived communication during the CT examination. There was no significant difference between both scanners (PCCT 4.77 ± 0.5 versus SCT mean 4.93 ± 0.3, *p* = 0.075).

Of all patients who were examined with the SCT, 100.0% stated that the communication during the CT examination was good or very good, whereas no patient found that the communication during the CT examination was bad or very bad (mean 4.93 ± 0.3, *p* = 0.075 compared to PCCT, see also Figure 9).

**Figure 10:**
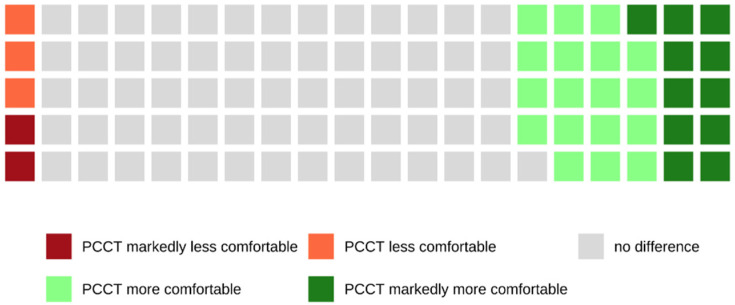
Waffle chart showing the comparison of both CT scanners in terms of communication. Most patients (66.1%) did not perceive a difference between both scanners, and only 28.6% of patients perceived the examination with the PCCT as more or markedly comfortable.

Of all patients who were examined with the PCCT and who were able to make a comparison between both scanners because of their previous CT examinations with the SCT, 28.6% rated the examination with the PCCT as more or markedly more comfortable, whereas 66.1% of these patients did not notice a difference between both scanners (see also Figure 10).

**Figure 11:**
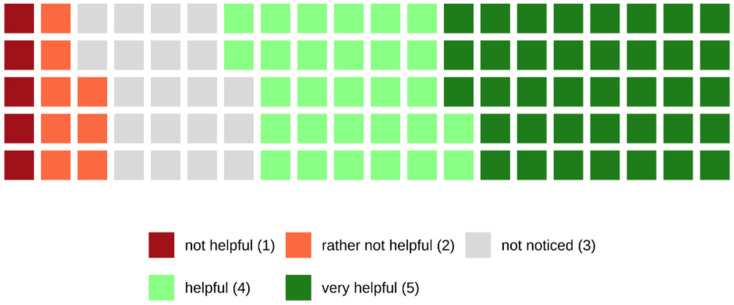
Waffle chart showing the answers regarding the helpfulness of the visual patient instructions. Most patients (67.0%) found the visual patient instructions helpful or very helpful.

Finally, 67.0% of the patients who were examined with the PCCT found the visual patient instructions helpful or very helpful, while 13% of the patients found the visual patient instructions rather not helpful or not helpful at all. The remaining 20% did not recognize the visual patient instructions during the examination (see also Figure 11).

## 4. Discussion

The present study evaluated the patient comfort during CT examinations performed with a modern, recently introduced, newly developed CT scanner (PCCT). Furthermore, a comparison of the perceived patient comfort during CT examinations with a standard CT scanner was made.

Overall, the surveyed patients were pleased with the comfort during CT examinations with both CT scanners. The survey on the standard CT scanner (SCT) did not reveal any significant deficits in terms of comfort. Thus, one could argue that the additional and expensive comfort-related features of a modern CT scanner do not matter. However, it is difficult to compare the benefit and costs of specific features of the PCCT, as certain characteristics of the scanner are not exclusively comfort-related but also influence the image acquisition (e.g., the speed of the CT table). Moreover, the survey showed that, beyond the decisive rating of single features, a large number of patients noticed a difference and perceived the new scanner to be more comfortable. In this context, the patients’ statements regarding comfort were of interest. 

The majority of patients who were interviewed after an examination with the standard CT as well as with the PCCT in our study had CT examinations in the past. Therefore, most patients included in this study were not inexperienced in terms of CT examination. Consequently, anxiety and inner tension that patients might feel undergoing their first CT examination should not have a major influence on the results of this study.

The diameter of the gantry of the PCCT is slightly bigger compared to the gantry of the SCT scanner. However, at the same time, the gantry of the PCCT is markedly deeper. Therefore, it was interesting to evaluate the patients’ feelings in terms of claustrophobia. The large majority of patients did not experience claustrophobic feelings during the examinations (PCCT 99.1%, SCT 93.2%), although a larger number of them found themselves to be claustrophobic in general. Many patients perceived the PCCT as more spacious and less oppressive. Patients stated that they did not notice the difference in depth of the gantry. Furthermore, patients perceived the PCCT to be not as loud as the SCT and, therefore, as less frightening.

In contrast to standard CT scanners, the CT table of the PCCT can be moved particularly low down to a minimum height of 38 centimeters, in order to make it easier for the patient to lie down and get up. However, the statements about the effort of lying down and getting up from the CT table in the present study show only a slight difference in favor of the PCCT—88.1% of the patients declared that it was easy or very easy after their examination with the PCCT (SCT 77.5%), whereas 11.9% found it difficult or very difficult (SCT 22.5%). When comparing both CT scanners, the majority of patients did not notice a difference. Interesting in this context is the statement of patients that the height of the CT table is less important while the gentle helping hand of the radiographer was found to be most valuable. Additionally, it was noticeable that the minimum height of the CT table was rarely fully utilized in practice.

The maximum speed of the CT table of the PCCT is markedly higher compared to standard CT scanners, which could potentially cause discomfort. In the clinical routine, the average scan time is a good surrogate parameter for the actual speed of the CT table during a CT examination. In the present study, the average scan time of the standard CT scanner was more than twice the length of the average scan time of the PCCT (see also Table 1). Nevertheless, 98.2% of the patients declared that the speed of the CT table of the PCCT was in a comfortable range. In fact, 46.0% of the patients rated the examination with the PCCT as more comfortable compared to the standard CT scanner (SCT), while 50.8% did not notice a difference between both scanners. This was often reasoned with the statement that the CT table of the PCCT moves more smoothly and evenly. The actual speed seems to be less decisive for the comfort of the patients. However, it has to be noted that the maximum speed of the CT table is used in only a few CT protocols.

The average scan time is not only a good surrogate parameter for the actual speed of the CT table but also approximates the time patients have to hold their breath during a CT examination. This time is markedly shorter for examinations with the PCCT (see Table 1) in comparison with the standard scanner (SCT). 

This is also reflected in the responses to our survey. Only 2.0% of all patients examined with the PCCT declared that the time in which they had to hold their breath was too long, whereas 20.7% of the patients examined with the SCT found that the time was too long. When comparing both CT scanners regarding breath-holding comfort, 38.6% of patients rated the examination with the PCCT as more comfortable. 

However, some patients state that it is rather the spacious feeling in the gantry that makes it easier to hold their breath than the actual time. Other patients mentioned that the visual patient instructions were distracting and may take away the uncertainty of how long they have to hold their breath.

In addition to the well-established audible instructions for breathing that patients receive during CT examinations, the PCCT offers visual patient instructions by showing simple pictograms on two small monitors inside the gantry. However, the present study reveals that audible instructions are probably sufficient to convey the instructions for breathing. No patient rated the communication during their examination with the SCT scanner as bad or very bad. Nevertheless, when comparing both scanners, 28.6% of the patients rated the examination with the PCCT as superior. This might be due to the fact that patients perceived the PCCT to be not as loud as the SCT.

Furthermore, 67.0% of the patients who were examined with the PCCT found the visual patient instructions helpful or very helpful. To some extent, this might result from the attention being drawn to the pictograms on the screens, which is likely to distract anxious patients from the scanning procedure itself.

The remaining 13.0% of the patients found the visual patient instructions rather not or not helpful at all, and 20.0% did not even recognize the visual patient instructions during the examination. The criticism expressed by the patients referred to the positioning of the screens. Some patients stated that the screens are too far away. Others explain that they generally keep their eyes closed during the examination and consequently do not profit from the visual patient instructions.

This study has limitations that need to be considered. Firstly, the number of patients interviewed was higher on the new scanner than on the standard scanner. Secondly, patients with experience in terms of CT examinations as well as patients undergoing their first CT examination were included in the analysis of this study. Therefore, not all patients were able to compare both CT scanners. Thirdly, no differentiation was made in terms of CT protocols, e.g., not every patient was asked to hold the breath during the CT examination.

It should be noted that the patient comfort-related features of the PCCT evaluated in the present study are not necessarily unique to this scanner. Other modern CT scanners might also offer these features.

Despite the abovementioned limitations, the present study provides first insights into the relevant aspects of patient comfort during CT examinations. Certain properties of a CT scanner, e.g., the speed and comfort of the CT table as well as the time in which patients have to hold their breath during the CT scan, seem to be important aspects contributing to the perceived patient comfort. Other features of the modern CT scanner, e.g., the visual patient instructions that are intended to support the communication during the CT scan, are generally also perceived positively but do not appear to be essential for patient comfort. 

Research has to be continued to understand the needs of patients in more detail and to further improve the patient comfort during radiological examinations.

## Figures and Tables

**Table 1:** Structural properties of both CT scanners that were used in this study. * patient height 175–185 cm, single contrast phase.

	Naeotom Alpha (PCCT)	Somatom Sensation 40 (SCT)
minimum height of the CT table	38 cm	53 cm
maximum speed of the CT table	737 mm/s	109 mm/s
gantry opening	82 cm	70 cm
average scan time (chest + abdomen CT) *	approx. 5 s	approx. 12 s
instructions	audible and visual	audible

## Data Availability

The data are available from the corresponding author on reasonable request.
